# Brain‐Wave Music Enhances Inhibitory Control Performance by Modulating Oscillations in the Motor Cortex and Frontoparietal Network

**DOI:** 10.1155/np/9433158

**Published:** 2026-09-28

**Authors:** Yan Li, Xinjian Su, Shenxin Hu, Liju Wang, Haoyu Bian, Chenxi Qiu, Haohan Yang, Hua Yang, Dezhong Yao, Jing Lu

**Affiliations:** ^1^ The Clinical Hospital of Chengdu Brain Science Institute, MOE Key Lab for Neuroinformation, University of Electronic Science and Technology of China, Chengdu 611731, Sichuan, China, uestc.edu.cn; ^2^ School of Life Science and Technology, Center for Information in Medicine, University of Electronic Science and Technology of China, Chengdu 611731, Sichuan, China, uestc.edu.cn; ^3^ Yingcai Honors College, University of Electronic Science and Technology of China, Chengdu 611731, China, uestc.edu.cn; ^4^ Department of Composition, Sichuan Conservatory of Music, Chengdu 610021, Sichuan, China, sccm.cn; ^5^ Key Laboratory for Music Digital Intelligence of Sichuan, Sichuan Conservatory of Music, Chengdu 610021, Sichuan, China, sccm.cn

**Keywords:** brain-wave music, inhibitory control, neural entrainment, personalized intervention

## Abstract

Inhibitory control modulation represents an important goal in neuromodulation research, with increasing attention to natural approaches such as music. However, personalizing music‐based interventions remains challenging. Brain‐wave music (BWM) provides personalized auditory stimulation by matching the oscillatory activity of brain waves. In this study, we investigated whether BWM—synthesized from an individual’s own neural oscillations—could effectively enhance inhibitory control. A total of 72 healthy participants participated in the inhibitory control function regulation experiment, with four types of musical stimuli used in the music intervention. The Go/Nogo task and electroencephalogram (EEG) recordings were administered before and after the music intervention to assess changes in inhibitory control function. We showed that a brief exposure to such personalized music enhanced participants’ performance on an inhibitory control task. In contrast, other forms of music intervention showed no significant effect. The intervention modulated theta oscillations in frontal and parietal brain regions, and these neural changes persisted beyond the stimulation period. Time‐resolved tracking revealed that frontal attentional control regions guided the entrainment of motor‐related areas. Furthermore, the alignment between the music and the individual’s intrinsic neural patterns correlated with behavioral improvement. These outcomes support the potential of BWM as a personalized auditory tool for cognitive enhancement in applied and clinical settings.

## 1. Introduction

Inhibitory control, a class of top‐down regulatory processes critical for adaptive behavior, is fundamentally implemented by the frontoparietal control network [1]. This system specializes in the continuous operations of conflict monitoring and inhibitory control, effectively suppressing extraneous stimuli and emotional distractions to facilitate focused task execution [2]. Frontal lobe damage or exposure to extreme work environments (e.g., space stations) can lead to decreased inhibitory control [3, 4]. Neurophysiologically, the functional integrity of inhibitory control is dependent on theta‐rhythmic oscillations originating in the frontal midline (FM) region. Electroencephalographic (EEG) research posits that this FM‐theta activity constitutes a vital endogenous neural mechanism underpinning attentional monitoring and the resolution of cognitive conflict, with its spectral dynamics serving as a robust predictor of behavioral efficacy [5, 6]. Therefore, the targeted modulation of FM‐theta rhythms has emerged as a rational and potent neuromodulatory approach for the enhancement of cognitive control [7].

Neural entrainment to naturalistic stimuli provides a potent pathway for the targeted modulation of neural oscillations [8]. This form of entrainment operates through a core mechanism: the temporal patterns of sensory input directly synchronize endogenous brain oscillations via phase resetting [9, 10]. This synchronization, quantified as oscillatory power in EEG signals, embodies a rhythmic fluctuation in neuronal excitability that is mechanistically linked to processes like selective attention [11]. It is a self‐sustained neural oscillatory process in the brain rather than a superposition of event‐related potentials [12], and the neural oscillation rhythm associated with the stimulus persists for a certain period after the stimulus has ended. As a supramodal self‐sustaining process, it is exceptionally well‐suited for sustained cognitive enhancement [13]. Consequently, entrainment via natural stimuli presents a unique alternative to methods like TMS, with auditory and visual modalities offering exceptional promise for translational applications due to their inherent practicality and ecological validity [14–16].

Music represents a particularly potent and ecologically valid stimulus within this neuromodulatory framework. The perception of music constitutes a canonical form of neural entrainment [17], wherein low‐frequency (*δ*, *θ*) cortical oscillations phase‐lock to the rhythmic structure of the acoustic input [18], reflecting the processing of various levels of musical rhythm structures [19]. Critically, this entrainment recruits a broad network that encompasses not only auditory areas but also the premotor and parietal cortices via the dorsal auditory stream [20]. This engagement generates a synchronized auditory‐motor response, which forms the neural basis for beat perception and sensorimotor integration [21]. Notably, the neural circuits engaged by music—particularly those involved in motor control and temporal prediction—significantly overlap with the frontoparietal inhibitory control networks. This convergence provides a foundational premise for music‐based interventions [22, 23], explaining their demonstrated potential in improving motor coordination [24] and facilitating the recovery of inhibitory control function [25].

However, the efficacy of natural sensory stimuli in neuromodulation is variable [26–28], a limitation particularly noted in music‐based auditory interventions. A primary source of this variability originates from individual differences in endogenous neural oscillatory patterns—a factor that prevailing intervention models have not adequately accounted for [14]. Indeed, the neural structure and function of the brain can vary significantly between individuals [29], and these differences may influence individual behavior in different ways [30]. This underscores the necessity for personalized intervention approaches, which are garnering increasing attention [31]. The effectiveness of entrainment is contingent on the specific characteristics of the brain’s oscillatory activity. For instance, perception is governed by rhythmic fluctuations of attention [32], and these individual differences in endogenous oscillations give rise to distinct patterns of information processing [33]. During entrainment, low‐frequency phasic entrainment to external stimuli correlates with fluctuations in the brain’s internal process state [34]. Thus, aligning external rhythmic stimulation with the intrinsic properties of an individual’s neural oscillations represents a promising strategy for achieving outcomes in cognitive enhancement [35].

The development of personalized music interventions is fraught with the challenge of profound inter‐subject variability in evoked neural responses [36, 37], driven by individual differences in musical training [38], language habits [39], and mental health [40]. In contrast, brain‐wave music (BWM), generated from an individual’s EEG, offers personalized musical stimulation that circumvents the confounding effects of individual variability. This translation is scientifically grounded in the shared power‐law dynamics—a scale‐free pattern, meaning that both music and spontaneous EEG exhibit similar power‐law distributions *S*(*f*) ~ 1/*f* ^β^ across different scales [41], which is believed to be a fundamental reason for music’s perceptual appeal [42]. BWM generates music that conforms to the patterns of spontaneous brainwave oscillations in individual brains using power‐law mapping. By listening to this musical representation of their own brain activity, individuals receive personalized intervention that modulates low‐frequency oscillatory activity. The effectiveness of BWM in cognitive function interventions has been previously validated in sleep regulation [43] and pain interventions [44]. Developing individualized treatment plans has been a major technical challenge in the treatment of psychiatric disorders [45, 46]. BWM, personalized entrainment stimulation based on patterns of neural oscillations in the brain, is a potential solution. Compared to other forms of stimulation, BWM offers a non‐invasive, low‐cost intervention that is easy to implement at home and can generate different types of music based on an individual’s neural oscillation characteristics.

We hypothesized that a personalized neural entrainment paradigm, using acoustic stimuli derived from participants’ own EEG oscillations, would cross‐modally modulate activity in inhibitory control networks and improve the behavior. To test this, we induced cognitive load and then presented personalized BWM. The study included three control conditions: non‐personalized music, classical music, and a no‐contact group. We assessed the inhibitory control function using the Go/Nogo task and concurrent EEG recordings.

## 2. Methods

### 2.1. Participants

Our study initially included 76 healthy participants (34 males and 42 females) from the University of Electronic Science and Technology of China. Two participants did not complete the task, and two were unable to finish the experiment due to a computer program issue. Ultimately, 72 participants (32 males and 40 females) were included. All participants were healthy, with no brain diseases and no medication taken in the past 3 months. Additionally, all of them were right‐handed, as assessed by the Edinburgh Handedness Inventory [47]. The psychometric tests included the following [48]: (i) Basic Information; (ii) Self‐rating Anxiety Scale (SAS); (iii) Self‐rating Depression Scale (SDS) [49]; (iv) Montreal Battery of Evaluation of Musical Abilities (MBEMA) [50]; (v) Edinburgh Handedness Inventory; and (vi) Barcelona Music Reward Questionnaire (BMRQ) [51]. MBEMA and BMRQ were used to distinguish differences among participants regarding their past sensitivity to music. All participants were randomly assigned to four groups (personalized, non‐personalized, classical music, and control). No significant differences in demographics were found among the four groups (see Table 1 for details).

**Table 1:** Participant demographics.

Characteristic	Personalized	Non‐personalized	Classical music	Control	*p*‐Value
Age (years)	21.83 ± 2.45	21.85 ± 2.21	21.89 ± 2.13	22.06 ± 1.83	0.98
SAS	27.00 ± 3.29	29.56 ± 7.98	28.50 ± 8.51	28.56 ± 5.43	0.71
SDS	44.11 ± 4.72	44.83 ± 4.68	44.22 ± 6.47	46.22 ± 4.67	0.59
BMRQ	76.22 ± 7.70	74.39 ± 5.86	73.39 ± 8.17	76.44 ± 9.36	0.59
MBEMA	13.72 ± 2.19	13.15 ± 2.41	13.78 ± 2.21	13.89 ± 2.47	0.75

*Note*: The survey of basic information and musical experience among the four groups of participants revealed no significant differences between them.

### 2.2. Procedure

All participants were asked to attend two experiments within a 5‐day period. The first experiment involved collecting basic participant information, questionnaires, and 10 min of resting‐state EEG data for BWM generation. Participants in the second experiment were instructed to wear an EEG cap and complete the entire procedure. Testing (EEG and psychometric tests) was carried out sequentially in the lab and lasted 60 min. All procedures occurred in the same soundproof room with controlled ambient noise to ensure consistency. Figure 1 shows the entire experimental session. Participants in each group had a 5‐min resting‐state EEG recorded at the beginning of the experiment (baseline). Then, participants were asked to complete a cognitive load task, which required them to remain attentive to the processing of visual or auditory information and to guide their finger presses. This task was intended to induce a temporary decline in inhibitory control by inducing cognitive fatigue. After the task, participants received a 10‐min music intervention or eyes‐open rest, depending on their subgroup. The Go/Nogo test assessed changes in participants’ inhibitory control function at baseline (Test1), pre‐intervention (Test2), and post‐intervention (Test3), as shown in Figure 1a. The Go/Nogo test included 300 trials (with 210 Go trials), and participants practiced for 30 trials before starting the experiment to acclimate to the test.

**Figure 1:**
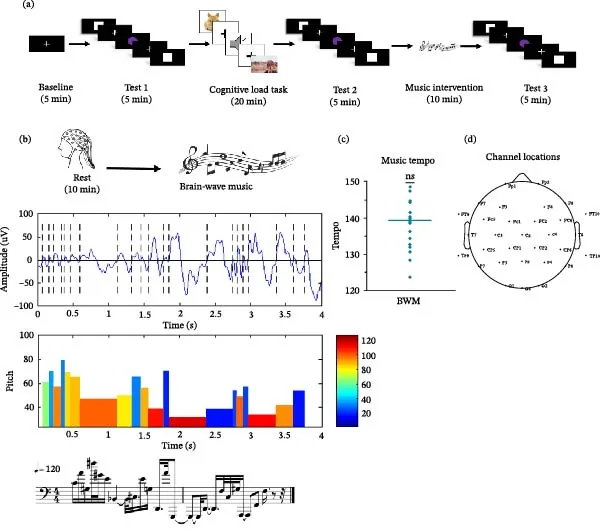
(a) Entire experimental procedure. The 10‐min music intervention (personalized, non‐personalized, classical music, or no‐contact) was scheduled after a 20‐min cognitive load task. The Go/Nogo test was done before the cognitive load task, after the cognitive load task, and following the musical intervention. (b) BWM was generated from 10‐min resting‐state EEG signals recorded from each participant within the BWM group, using the electrode at FCz [41]. (c) The musical tempo of CM was not significantly different from BWM. Each point in the figure represented a piece of BWM, and the *Y*‐axis represents the calculated tempo value of the music. (d) The EEG cap with 32 Ag/AgCl electrodes positioned according to the International 10–20 system, and the ‘Fz’ electrode served as the reference.

### 2.3. Music Materials

The algorithm for generating BWM drew upon our team’s previous work [41]. This algorithm combines the scale‐free properties that EEG signals and music share and can fit the power‐law features of EEG [52, 53]. BWM’s temporal structure directly maps amplitude envelopes to melodic contours and instantaneous frequency to rhythmic density, creating isomorphic representations of neural dynamics. The note pitches of BWM follow the mapping relationship:

pitch=−cαlgF+h,

where *h* and *c* are constant and *α* is a power‐law index of the EEG. The frequency *F* is proportional to the amplitude (peak‐to‐peak) of the EEG signal. The note duration of the BWM is equal to the duration between the two over‐zero points of the EEG signal.

tnote=tEEG.

The musical intensity (MI) of the notes is then distributed according to the following pattern:

MI=k lgAP+l,

where AP is the average power of the EEG signal and *k* and *l* are constants. Specific steps can be found in a previous work [41].

In this study, BWM was created from 10‐min resting‐state EEG signals recorded at the FCz electrode, which reflected activity in the main brain regions responsible for controlling motor behavior [54]. All BWMs were generated from resting‐state EEG signals acquired by participants in the personalized group in the first experiment. During acquisition, participants were instructed to keep their eyes open, clear their minds, and refrain from performing any cognitive activity. To modulate participants’ theta oscillatory activity, a band‐pass filter was used between 4 and 8 Hz, and the averages were re‐referenced before being converted to music. Music generation was achieved using the MIDI toolbox in MATLAB. Participants in the non‐personalized group also listened to BWM, but it did not originate from their own brainwave signals. Based on their subjective reports, each participant generated a BWM that sounded similar to a piano piece composed by a composer. Classical music samples were selected from the Chinese Affective Music Store (CAMS).

The MIR toolbox was used to compute the actual rhythms of the two types of music. This calculation was performed using the MIR Toolbox to analyze the acoustics of the music clip. The actual tempo of the music clip was estimated by extracting the start points of musical events and analyzing the autocorrelation of the audio start events. In this study, the estimated actual tempos for both BWM and classical music were around 140 BPM. A one‐sample *t*‐test comparison showed that there was no significant difference between BWM and classical music (*t* = 1.120, *p* = 0.277), which prevented discrepancies in neural entrainment caused by the actual rhythm of the music.

### 2.4. Cognitive Load Task

The cognitive load task was designed based on the intrinsic cognitive load involved in the human brain’s information processing. Participants in this session were instructed to press the corresponding keys on the keyboard in response to stimuli presented on the computer. The experimental stimuli comprised both visual and auditory components. The auditory stimuli consisted of single syllables at 1000 and 2000 Hz, with 20 and 30 dB, corresponding to the keys 1, 2, 9, and 0 on the keyboard. Visual stimuli were presented as images. When participants saw an image, they were instructed to determine whether the content depicted a human, animal, plant, or non‐living object and press the corresponding key on the keyboard: 1, 2, 9, or 0. Each stimulus was presented at 2‐s intervals, with visual and auditory stimuli presented in a mixed sequence. The entire paradigm lasted 20 min. Before the experiment, participants were familiarized themselves with the types of stimuli that might appear, and their ability to distinguish between them was confirmed.

### 2.5. Go/Nogo Test

The Go/Nogo test was presented as a visual stimulus. Participants were instructed to press the “g” key on the keyboard within 500 ms when a white square pattern appeared while refraining from pressing any keys when other patterns were displayed. To increase the difficulty of inhibiting control behavior and to assess participants’ ability to control hand movements, each test comprised 210 Go trials and 90 Nogo trials, for a total of 300 trials. The order of each trial was completely randomized, with a presentation interval of 1000 ms and a presentation duration of 500 ms. In this study, participants’ response correctness across all trials and reaction time (RT) in Go trials were used as a behavioral assessment of inhibitory control.

### 2.6. EEG Recording

EEG was recorded using an active electrode system (ActiCap, Brain Products, Gilching, Germany). The participants wore an EEG cap with 32 Ag/AgCl electrodes positioned according to the International 10–20 system, and data were sampled at 1000 Hz. The “Fz” electrode served as the reference. Electrode impedances were maintained below 5 kΩ. The neuroelectric activity was then stored for offline analysis.

### 2.7. EEG Preprocessing

Offline processing of the EEG data was performed in MATLAB (R2014a; MathWorks, Natick, MA, USA) using the EEGLAB toolbox [55] and Brainstorm software [56]. Then, the data were referenced to the average of all electrodes by using the EEGLAB toolbox. Continuous EEG data were high‐pass filtered above 1 Hz, low‐pass filtered below 100 Hz, and notch‐filtered at 49–51 Hz to remove industrial AC frequencies. Subsequently, the EEG data were segmented into epochs spanning from 200 ms before to 800 ms after stimulus onset, followed by baseline correction using the pre‐stimulus interval. Time‐locked averaging was applied to enhance the signal‐to‐noise ratio as the spontaneous background EEG is nonlinear, nonstationary, and typically larger in amplitude than the ERP signal. Subsequently, independent component analysis was used to identify and remove ocular artifacts associated with eye blinks and movements. After ocular correction, all trials were screened for additional artifacts; epochs containing deflections exceeding ±100 µV were marked and excluded from further analysis [48].

### 2.8. Time–Frequency Analysis

Time–frequency analysis was implemented to identify brain oscillations in Go/Nogo tests. As this study focused on inhibitory control, we selected typical electrodes in the FM, including FC1, FC2, and Cz, which are associated with the central executive process in neuroimaging studies. After that, we focused on theta band oscillations (4–8 Hz) as they have been shown to relate to inhibitory control [5, 57, 58]. Additionally, since the behavioral results indicated an average RT of ~300 ms, we selected the 150–450 ms interval to examine prior motor neural oscillations. Specifically, a time–frequency distribution (TFD) of the EEG signal was generated using the Morlet wavelet transform. The wavelet family was defined by a Gaussian‐shaped kernel in both time and frequency domains, providing a trade‐off between temporal and spectral resolution. During this analysis, the EEG signal was considered stationary only over short intervals. For each point on the time–frequency plane, a complex time–frequency estimate, *F*(*t*,*f*), was derived using the Morlet wavelet transform. The analysis covered a time window from −200 ms to 800 ms (in steps of 10 ms) and a frequency range of 0.1–40 Hz (in steps of 0.1 Hz). Signal power at each corresponding time–frequency point was then quantified by the power spectrum *P*(*t*,*f*) = |*F*(*t*,*f*)|^2^, which provides a joint time–frequency representation. This process yielded a TFD of the signal [48].

### 2.9. Power Spectral Density (PSD) Analysis

BWM generated from theta EEG could modulate the listener’s theta oscillations, which we analyzed by PSD. To observe the theta oscillatory activity of the brain and eliminate the interference of 1/*f* noise in the EEG spectrum, we used the time‐resolved spectral parameterization (SPRiNT) method [59, 60], which accomplished the modeling and analysis of the time‐dependent 1/*f* spectrum after doing a short‐time Fourier transform of the EEG signals, and the Brainstorm Toolbox [56] performed this process. The settings for the SPRiNT algorithm, which has been shown in previous studies to be effective in isolating theta oscillatory activity in the cerebral cortex, were set as follows. The power spectra were parameterized across the frequency range of 2–20 Hz to obtain the peak frequency and amplitude in the theta frequency range. The peak width limits were set at 0.5–2 to identify frequency‐specific peaks. The maximum number of peaks was set at 4, assuming that there could be four meaningful peaks in the 2–20 Hz frequency range, that is, one in each band (delta, theta, alpha, and beta). No minimum peak height was set; the peak threshold was set at 2, and the aperiodic mode was fixed. The short‐time Fourier transform used a window length of 10 s and a 50% window overlap [61]. SPRiNT was averaged over time in either the intervention or baseline sessions. Among other things, we focused on changes in FM areas (FC1, FC2, and Cz) related to inhibitory control functions, parietal cortex (P3 and P4), and auditory cortex (T7 and T8).

### 2.10. Phase Locking Value (PLV) Analysis

Synchronized activity between brain regions is a crucial approach for investigating the properties of brain networks and distinguishing different mental or cognitive activities. PLV was commonly used for studying functional connectivity and was adopted to capture the phase synchronization between paired nodes in Go or Nogo trials [62]. Processing was performed using Brainstorm software. In the Go/Nogo test, we computed the functional connectivity of a motor network consisting of FC1, FC2, P3, and P4 electrodes, which were significantly activated in inhibitory control functions in previous studies [63]. We computed functional connectivity within the network separately based on the PLVs that the Go and Nogo trials elicited. Then, the difference matrix between the Nogo and Go trials was obtained by subtraction.

During the music intervention, we calculated network connectivity of the frontoparietal network—comprising the frontal (F3 and F4) and parietal (P3 and P4) lobes—and the midfrontal region (FC1, FC2, and Cz) with the frontal, parietal, and auditory cortices.

### 2.11. Fitting of BWM to EEG

The fitting of BWM to EEG rhythms was an essential factor enabling personalized modulation. The degree of fitting indicated whether the BWM we generated truly fitted with the participant’s spontaneous EEG activity. Therefore, we analyzed whether the pitch direction of BWM accurately reflected the fluctuating trend of EEG signals. The relationship between the pitch of the BWM and the amplitude of the EEG conforms to a logarithmic mapping (*pitch~-lg Amp*) [41]. We segmented the EEG signal and BWM using time windows. We calculated the amplitude of the EEG signal and the average pitch of the BWM within each time window. The degree of fitting between EEG and BWM (BWM–EEG fitting, BEF) for each piece of music was obtained by calculating Pearson’s correlation coefficient between the logarithm of the amplitude of the corresponding segment and the mean pitch. Since the coefficient *m* < 0, we multiplied the calculated Pearson’s correlation coefficient by −1. This way, the closer the BEF is to 1, the more similar the pitch direction of the BWM is to the amplitude fluctuations of the EEG.

### 2.12. Statistical Analysis

In this study, we used a two‐way analysis of variance (group × stage) to examine behavioral performance and EEG pattern changes across different stages of the experiment among participants from various groups. As an exploratory study, to maximize outcome discovery, we employed paired *t*‐tests (*n* = 18) or repeated measures ANOVA (*n* = 54) to analyze within‐group differences in behavioral and EEG characteristics across the stages for each participant group. For repeated within‐group comparisons, statistical results were corrected using the FDR or Bonferroni methods. Additionally, this study used Pearson correlation analysis to explore the relationship between EEG features and behavioral performance or musical elements.

## 3. Results

### 3.1. Participants in the Personalized Group Exhibited Significant Improvement in Inhibitory Control Following the Musical Intervention

The Go/Nogo test was used in this study to assess participants’ inhibitory control functions by analyzing the effects of different interventions. A two‐way ANOVA was conducted to analyze participants’ accuracy rates and RTs. Results revealed significant main effects of time (research sessions) on both accuracy (accuracy: *F*_sessions_ = 20.09, *p*_sessions_ < 0.001; *F*_group_ = 1.39, *p*_group_ = 0.25; *F*_sessions × group_ = 0.78; *p*_sessions × group_ = 0.58) and RT (RT: *F*_sessions_ = 14.54, *p*_sessions_ < 0.001; *F*_group_ = 1.41, *p*_group_ = 0.24; *F*_sessions × group_ = 1.795; *p*_sessions × group_ = 0.10), with no significant differences observed between groups. To further clarify these differences, a repeated‐measures ANOVA was used to examine each group’s Go/Nogo test scores across different research sessions.

Figure 2 shows the mean accuracy and RT of the four groups of participants at different stages of the Go/Nogo test. Repeated‐measures ANOVA revealed that participants in all four groups showed significant differences in accuracy (personalized: *F* = 7.896, *p* = 0.002; non‐personalized: *F* = 4.149, *p* = 0.035; classical music: *F* = 6.588, *p* = 0.009, control: *F* = 5.550, *p* = 0.012) and RT (personalized: *F* = 4.361, *p* = 0.024; non‐personalized: *F* = 3.852, *p* = 0.034; classical music: *F* = 5.550, *p* = 0.008, control: *F* = 6.680, *p* = 0.009) between sessions of the experiment (Figure 2a). Post hoc analysis showed that all four groups of participants showed a significant decrease in accuracy after the cognitive load task (personalized: *t*_2vs1_ = −2.812, *p* = 0.036; non‐personalized: *t*_2vs1_ = −2.759, *p* = 0.040; classical music: *t*_2vs1_ = −3.013, *p* = 0.023; control: *t*_2vs1_ = −2.760, *p* = 0.040, two‐tailed, all Bonferroni corrected). After the 10‐min music intervention, accuracy was significantly greater in the personalized group than pre‐intervention (*t*_3vs2_ = 4.203, *p* = 0.001), whereas there was no significant increase in the other three groups (non‐personalized: *t*_3vs2_ = 1.942, *p* = 0.206; classical music: *t*_3vs2_ = 2.545, *p* = 0.062; control: *t*_3vs2_ = 2.470, *p* = 0.073, two‐tailed, all Bonferroni corrected). All four groups of participants showed an overall decreasing trend in Go trial response times during the Go/Nogo tests (personalized:*t*_2vs1_ = 0.301, *p* > 0.999, *t*_3vs2_ = −2.669, *p* = 0.048, *t*_3vs1_ = −2.840, *p* = 0.033; non‐personalized:*t*_2vs1_ = −3.925, *p* = 0.033, *t*_3vs2_ = 1.949, *p* = 0.374, *t*_3vs1_ = −1.985, *p* = 0.361; classical music: *t*_2vs1_ = −2.226, *p* = 0.110, *t*_3vs2_ = −1.10, *p* = 0.98, *t*_3vs1_ = −3.095, *p* = 0.019; control: *t*_2vs1_ = −3.426, *p* = 0.009, *t*_3vs2_ = −1.001, *p* = 0.993, *t*_3vs1_ = −2.759, *p* = 0.040, two‐tailed, all Bonferroni corrected) (Figure 2b). The participants’ RTs did not show a significant prolongation following cognitive load.

**Figure 2:**
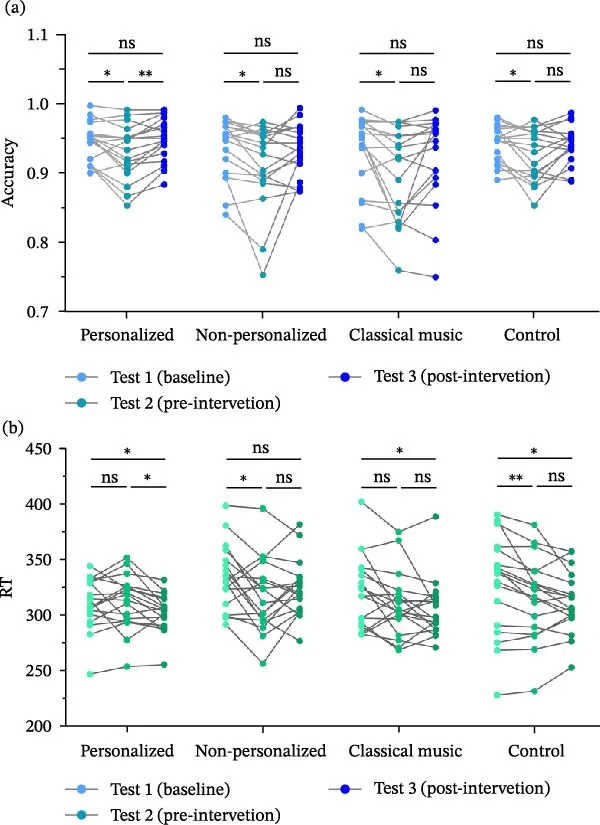
Reaction time (RT) and accuracy on the Go/Nogo test for four groups of participants. (a) Differences in accuracy across three Go/Nogo tests for each group of participants. (b) Differences between participants in each group during the Go trial response time in three Go/Nogo tests (ns *p* > 0.05,  ^∗^ *p* < 0.05,  ^∗∗^ *p* < 0.01, Bonferroni corrected).

### 3.2. FM Theta Oscillatory Activity During Inhibitory Control Among Participants in The Personalized Group Was Enhanced After The Music Intervention

To investigate whether the personalized intervention affected the brain’s inhibitory control functions, we examined the neural activity during the Go/Nogo task before and after the music intervention. Figure 3 shows differences in theta oscillatory activity and frontal‐parietal connectivity activation during inhibitory control in participants before and after the music intervention following the cognitive load task. The results of the paired *t*‐test showed that the FM theta power during inhibitory control (Nogo trials minus Go trials) for participants in the personalized group was significantly enhanced after the music intervention (*t* = 2.480, *p* = 0.023), while the other two groups showed no significant differences (non‐personalized: *t* = 1.042, *p* = 0.312; classical music: *t* = 0.114, *p* = 0.910; control: *t* = 0.217, *p* = 0.830) (Figure 3b). However, two‐way ANOVA did not reveal any significant main effects or interaction effects. (*F*_sessions_ = 1.782, *p*_sessions_ = 0.1864; *F*_group_ = 0.528, *p*_group_ = 0.664; *F*_sessions × group_ = 0.512; *p*_sessions × group_ = 0.674).

**Figure 3:**
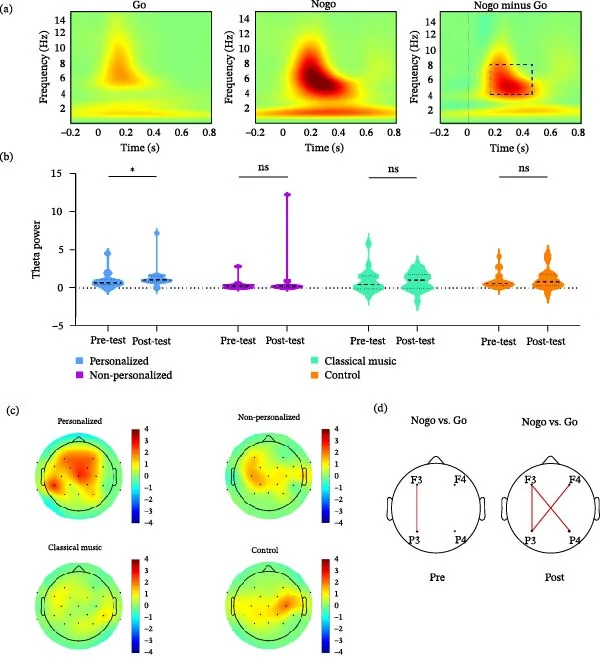
(a) Mean time–frequency power distribution of all participants before the musical intervention (Go/Nogo2). Theta (4–8 Hz) oscillatory activity during inhibitory control (150–450 ms) was mainly analyzed in this study (marked with a box in the figure). (b) Group mean difference in theta power between Go and Nogo trials at pre‐test (Test2) and post‐tests (Test3). The measurements reflect the average theta power (4–8 Hz) from the FM between 150 and 450 ms post‐stimulation (mean FC1, FC2, and Cz). (c) Changes in Theta power on the Go/Nogo test before and after the intervention for four groups of participants. (d) PLV‐based theta network activation between the prefrontal cortex and parietal cortex during inhibitory control (difference between Nogo trials and Go trials) in participants in the BWM group in the pre‐ and post‐tests (*p* < 0.05). The red line indicates connections with significantly higher PLV values in the Nogo trial than in the Go trial.

Furthermore, the activation of the frontoparietal network during inhibitory control plays a critical role in human cognitive control. We conducted paired *t*‐tests to examine network activation during personalized group participants’ inhibitory control in Nogo and Go trials and compared the differences before and after the music intervention. In the pre‐test, significantly higher PLV was found only in participants’ F3–P3 (*t* = 2.185, *p* = 0.043) connections. In the post‐test, significantly higher PLV was found in participants’ F3–P3 (*t* = 2.287, *p* = 0.035), F3–P4 (*t* = 2.125, *p* = 0.048), and F4–P3 (*t* = 2.401, *p* = 0.028) (Figure 3d).

### 3.3. Personalized BWM Neural Entrainment Modulated Participants’ Theta Oscillatory Activity in the FM and Parietal Cortex

Neural entrainment is the brain’s selective attentional behavior to external stimuli. We compared participants’ theta energy during musical interventions using power spectral analysis to explore the neural entrainment of different interventions to participants’ cortical theta oscillations. The SPRiNT algorithm excluded the interference of non‐periodic components during the analyses [64]. The regions we studied include the bilateral frontal, parietal, temporal, and prefrontal cortices. A paired *t*‐test was used to examine differences in theta oscillatory activity during music listening relative to baseline. The results are shown in Table 2. Electrodes (FC1, FC2, Cz, T7, T8, P3, P4, F3, and F4) were selected to target specific brain regions under investigation. No significant differences in baseline theta power were found among the four groups (*F* = 0.47, *p* = 0.701), ensuring comparability.

**Table 2:** Theta power during music intervention.

Group	Region	Electrodes	*p*‐Value	*t*‐Value
Personalized	Frontal midline	FC1	0.044 ^∗^	2.372
		FC2	0.035 ^∗^	2.585
		Cz	0.027 ^∗^	2.899
	Parietal cortex	P3	0.022 ^∗^	3.133
		P4	0.008 ^∗∗^	3.847
	Frontal cortex	F3	0.012 ^∗∗^	3.505
		F4	0.006 ^∗∗^	4.451
	Temporal cortex	T7	0.035 ^∗^	2.750
		T8	>0.999	1.369

Non‐personalized	Frontal midline	FC1	0.885	0.147
		FC2	0.017 ^∗^	2.637
		Cz	0.112	1.675
	Parietal cortex	P3	0.085	1.827
		P4	0.168	1.441
	Frontal cortex	F3	0.003 ^∗∗^	3.492
		F4	0.006 ^∗∗^	3.155
	Temporal cortex	T7	0.038 ^∗^	2.253
		T8	0.286	1.102

Classical music	Frontal midline	FC1	0.879	0.154
		FC2	0.936	0.082
		Cz	0.988	0.015
	Parietal cortex	P3	0.649	−0.463
		P4	0.764	−0.305
	Frontal cortex	F3	0.089	1.803
		F4	0.326	1.012
	Temporal cortex	T7	0.250	1.190
		T8	0.532	0.638

Control	Frontal midline	FC1	0.213	1.293
		FC2	0.191	1.362
		Cz	0.172	1.427
	Parietal cortex	P3	0.210	1.304
		P4	0.158	1.478
	Frontal cortex	F3	0.114	1.669
		F4	0.123	1.624
	Temporal cortex	T7	0.896	0.133
		T8	0.093	1.781

*Note*: Differences in theta oscillatory activity of participants during music intervention compared with baseline (ns *p* > 0.05, two‐tailed, and FDR corrected in personalized group).

^∗^ *p* < 0.05.

^∗∗^ *p* < 0.01.

Synchronized activity within the parietal‐frontal network is also a crucial factor influencing inhibitory control functions. A paired *t*‐test was employed to investigate the synchronized activity within this network during the music intervention. Significant synchronized activity was observed across the personalized (F3–P4: *t* = 2.427, *p* = 0.026; F4–P3: *t* = 2.903, *p* = 0.009, two‐tailed), non‐personalized (F3–P3: *t* = 2.150, *p* = 0.046; F4–P3: *t* = 2.636, *p* = 0.017, two‐tailed), and classical music (F3–P3:*t* = 2.809, *p* = 0.012; F4–P4: *t* = 2.167, *p* = 0.044, two‐tailed) groups throughout the process (Figure 4c). This phenomenon may be related to the involvement of the frontotemporal network in music processing. In the personalized group, we tested whether higher FM theta power during the intervention was associated with better behavioral performance by calculating the correlation between theta power and the Go/Nogo performance. Pearson’s correlation analysis showed a significant positive correlation between the theta power of the BWM group during the intervention and the accuracy of the Go/Nogo test at post‐test (*R* ^2^ = 0.322, *p* = 0.013) (Figure 4d).

**Figure 4:**
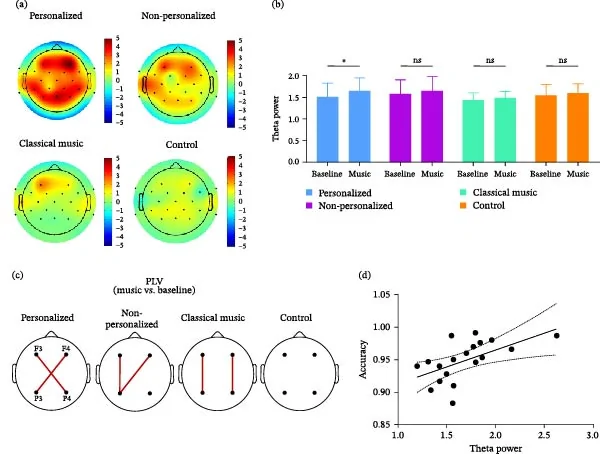
(a) Distribution of changes in theta oscillations in the cerebral cortex during music listening. (b) Differences in theta oscillations in FM regions during musical interventions. (c) PLV‐based network activation between the frontal and parietal regions during the music intervention (*p* < 0.05). The red line indicates connections with significantly higher PLV values in the intervention than in the baseline. (d) Correlation between the personalized group’s FM theta power and Go/Nogo accuracy (*R* ^2^ = 0.322, *p* = 0.013).

Additionally, we employed paired *t*‐tests to measure synchronized activity between the FM region and the bilateral frontal cortex, parietal cortex, and auditory cortex. Notably, electrodes in the FM region established significant connections with the right frontal lobe, left parietal lobe, and left auditory cortex (FC2–F4:*t* = 3.244, *p* = 0.031; FC2–P3: *t* = 3.681, *p* = 0.031; FC2–T7: *t* = 2.965, *p* = 0.031; Cz–F4: *t* = 3.031, *p* = 0.031; Cz–T7: *t* = 3.123, *p* = 0.031, two‐tailed, FDR corrected), which was not observed in the other three participant groups (Figure 5a).

**Figure 5:**
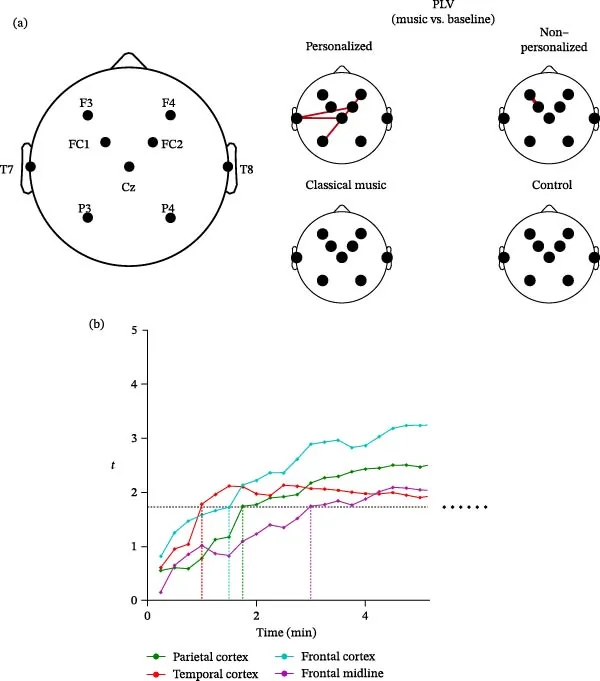
(a) We focused on synchronized activity between FM areas (FC1, FC2, and Cz) related to inhibitory control functions and frontoparietal network (F3, F4, P3, and P4) and auditory cortex (T7 and T8). During music listening, the midline regions of the personalized group participants showed significantly higher PLV values with the right frontal lobe, left temporal lobe, and left parietal lobe (*p* < 0.05, FDR corrected). The red line indicates connections with significantly higher PLV values in the intervention than in the baseline. (b) Significance analyses conducted during the intervention showed that significant theta changes were greatest in the temporal cortex, followed by the parietal and prefrontal cortex, and finally the premotor cortex. Theta oscillations in the frontal cortex, the temporal cortex, the parietal cortex, and the frontal midline showed significant enhancement within about 3 min.

The frontal cortex has the function of top‐down regulation of neural entrainment in the sensory cortex [65]. Compared to neural entrainment in the gamma band, the process of music‐based low‐frequency neural entrainment intervention requires more involvement of the higher cognitive cortex. In addition to its own rhythmic variations, the complexity of the music and the listener’s musical ability influence music‐based auditory neural entrainment effects—all of which reflect the fact that musical neural entrainment is modulated by the frontal cortex for musical cognitive behaviors [66–69]. Here, we investigated auditory entrainment in BWM using a time course analysis of significance during the intervention (Figure 5b). A significant enhancement in theta oscillations in the frontal and parietal lobes occurred prior to the premotor cortex. The chronological sequence in which theta oscillations became more pronounced reflects the order in which different cortical regions in the participants’ brains were affected during the personalized BWM intervention.

### 3.4. The Effect of BWM on Theta Oscillatory Entrainment Is Influenced by Its BEF

Compared to other forms of music, BWM intuitively reflects the electrophysiological characteristics of the human brain, and its pitch direction illustrates the fluctuating characteristics of neural oscillations. Fitting this fluctuating trend is an essential basis for BW to enable personalized modulation of individual neural activity characteristics. We reflected this property of BWM through a correlation index between BWM pitch and EEG signal amplitude (see Section 2 for details). Figure 6 shows the results of Pearson correlation analysis. We examined whether the personalized BWM intervention’s effect on participants’ inhibitory control is related to its characterization of EEG oscillation patterns. Correlation analysis revealed that both theta oscillatory activity between the Go/Nogo pre‐ and post‐tests (*R* ^2^ = 0.309, *p* = 0.016) and the theta oscillatory activation of the FM during music listening (*R* ^2^ = 0.241, *p* = 0.038) were significantly positively correlated with the degree of BWM fitting with the EEG (Figure 6b,c).

**Figure 6:**
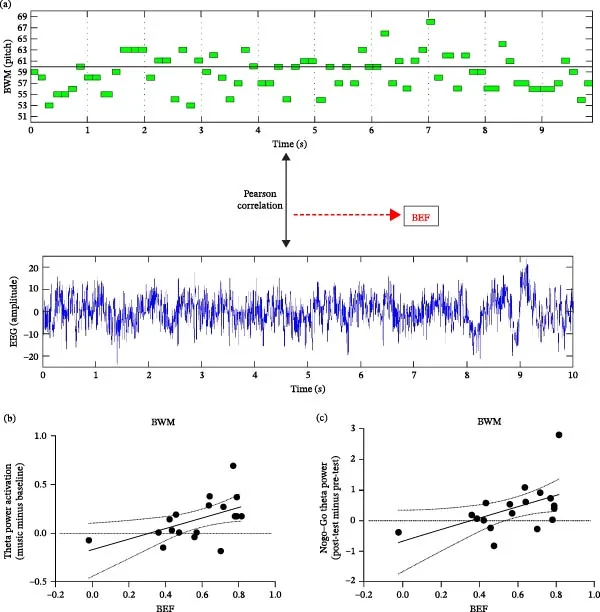
(a) BEF calculation. For each participant’s BWM, the correlation between its pitch variation direction and the corresponding EEG amplitude fluctuation is calculated to derive the BEF. (b) The BEF showed a significant positive correlation with enhancement of theta oscillations (music minus baseline) during the music intervention (*R* ^2^ = 0.309, *p* = 0.016). (c) The BEF influences theta oscillations (pre‐test minus post‐test) of FM in the Go/Nogo test before and after intervention (*R* ^2^ = 0.241, *p* = 0.038).

## 4. Discussion

This study demonstrates that personalized BWM offers a novel approach to enhancing inhibitory control through a music‐based intervention. A brief 10‐min BWM session effectively modulated cortical theta oscillations via neural entrainment, restoring inhibitory control abilities impaired by the cognitive load. We hypothesized that BWM can enhance inhibitory control by modulating the oscillations of neural structures common to both music processing and inhibitory control through the neural entrainment effect. Although between‐group differences were not statistically significant, within‐group improvements consistently supported the intervention’s efficacy. The premotor cortex, identified as a multimodal hub for music perception and motor control, served as the key target of this modulation. During the Go/Nogo test, this region exhibited significantly enhanced theta oscillatory activity and network connectivity following BWM intervention. Importantly, the intervention’s effectiveness depended on how well the music matched the participant’s neural patterns, with the prefrontal cortex playing a critical role in allocating cognitive resources to motor areas during musical stimulation.

The sustained theta entrainment driven by personalized BWM selectively enhanced cortical theta oscillations, an effect uniquely observed in the BWM group. This enhancement was detected not only in the FM region but also across a network including the left auditory cortex, bilateral prefrontal cortex, and parietal cortex. This phenomenon may reflect the unique qualities of personalized BWM. Theta enhancement in the left temporal cortex is linked to information processing during auditory entrainment. The phase of low‐frequency activity (<10 Hz) in the local network of the primary auditory cortex (A1) coincides with the external stimulus, and this local spike‐network coupling plays a role in encoding continuous auditory signal stimuli [70]. It essentially reflects the processing of a constant stream of external auditory information in the auditory cortex and is the basis for the brain’s ability to realize auditory entrainment [17, 71]. More importantly, during BWM intervention, theta entrainment in the FM brain regions involved in inhibitory control is also modulated. We observed that auditory neural entrainment drives theta oscillations in FM brain regions and synchronized oscillatory activity in the frontotemporal network. The FM brain area includes the supplementary motor area and premotor cortex of the cerebral cortex, and one of its prominent roles is integrating sensory information and controlling limb movement behavior [72, 73]. The prefrontal motor cortex is a potential neural mechanism for enhancing inhibitory control functions. This structure is also involved in music perception, particularly rhythm perception associated with entrainment [74, 75]. This “dual identity” enables us to modulate activity in this brain region by music [25]. Thus, neural entrainment modulates theta oscillations in the FM region and synchronizes activity in the frontoparietal network, which explains why BWM enhanced participants’ control behavior.

By comparing participants’ EEG and behavioral performance before and after the intervention, we found that the modulatory effects of BWM on FM areas and the premotor–parietal network persisted into the subsequent Go/Nogo task. Specifically, in the BWM group, FM theta oscillations during the music intervention showed a significant positive correlation with accuracy on the post‐intervention Go/Nogo task; moreover, synchronous oscillations between frontal and contralateral parietal lobes during the intervention also persisted into the post‐intervention test. PLV analysis further revealed differences in network connectivity between the premotor cortex and parietal cortex before and after the intervention. FM theta oscillations reflect the dynamic interaction between the prefrontal cortex and anterior cingulate cortices [76], serving as a crucial neural marker for human inhibitory control behaviors [77, 78]. The more pronounced theta oscillations observed in the Nogo trial compared to the Go trial reflect response inhibition [79] and conflict monitoring (selective attention) behaviors [6] during inhibitory control processes. Improvement in the Go/Nogo performance after a 10‐min BWM intervention indicates causal enhancement of response inhibition. Synchronous oscillations between the frontal and contralateral parietal lobes during the music intervention persisted into the post‐intervention Go/Nogo test. Synchronous oscillations between the frontal and parietal cortices serve as the neural basis for humans’ ability to control motor behavior properly, as demonstrated in studies of inhibitory control dysfunction in patients with ADHD [80] and Parkinson’s disease [81]. In addition, during active control processes in the brain, there is a significant increase in theta‐synchronous oscillations between the prefrontal and parietal cortices [82], typically serving as the primary target for inhibitory control‐related modulation [83–85]. These findings demonstrate that the supramodality of personalized BWM neural entrainment can be harnessed in cognitive interventions to modulate the neural oscillatory activity during task performance.

Matching music patterns with individual brain oscillation features forms the foundation for implementing personalized intervention programs. BWM generated based on the EEG 1/*f* power law distribution has a significant advantage over non‐personalized or classical music by accurately preserving EEG oscillatory features in the music. The low‐frequency variation of theta oscillations in spontaneous EEG plays a role in regulating attentional fluctuations in the human brain [86], which enables BWM rhythms to align with variations in the brain’s intrinsic cognitive rhythms [87]. Specifically, theta‐band activity in the higher cognitive cortex and primary auditory cortex plays a significant role in processes such as attentional selection and cognitive control [88]. For example, during the process of anticipating music, the frontoparietal network serves as the primary network responsible for cognitive control [89]. Synchrony in theta oscillations in the auditory cortex reflects selective attention during music listening [90, 91]. The related neural oscillatory activity (such as theta oscillations and synchronous activity) was modulated by personalized entrainment of the BWM. We observed that the BWM fitting to EEG signals was significantly and positively correlated with the effectiveness of the personalized intervention on inhibitory control functions. This result demonstrates that matching the external stimulus intervention to the intrinsic neural activity is an essential factor in the intervention’s effectiveness. Personalized music interventions need to align with each participant’s cognitive pattern, but more evidence is needed to understand how this pattern develops.

BWM is not just a passive acoustic stimulation method but also a cognitive processing involving the higher cognitive cortex. Our analysis of how the significance of neural effects changed over time during BWM stimulation indicates that the prefrontal and frontotemporal networks play a crucial role in the intervention. In the BWM group, significant theta oscillations were first detected in the left temporal cortex during the intervention. The left temporal cortex is the primary auditory cortex involved in perceiving the rhythm of music [92]. This is consistent with our design, where changes in theta oscillations during music intervention come from musical neural entrainment rather than other factors. Notably, following the temporal cortex, the frontal and parietal cortices exhibited significant changes first, rather than the premotor cortex, which is considered closer to the auditory cortex. Synchronized activity in the frontoparietal cortex is associated with the cognitive processes of music perception. Activity changes in the frontoparietal network were observed across all four groups of participants who received music intervention. This demonstrates that the modulatory effects of BWM on cortical oscillations are related to higher cognitive processes in the human brain. Frontal regions, such as the inferior frontal gyrus, dorsolateral prefrontal, and medial prefrontal, tend to show sustained increase in theta power during music, and this activation is associated with behaviors like emotional perception, encoding, prediction, or memory during music [75, 93–95]. A previous study found that prefrontal oxyhemoglobin concentrations and dopamine gene expression are altered during music listening, demonstrating that the frontal regions of the brain are involved in the processing of musical information [96–98]. The significant enhancement of theta oscillatory activity in the primary motor cortex during music (more >3 min after the start of the music) occurred later than those in the auditory cortex and frontotemporal regions (less <2 min after the start of the music). This temporal difference indicates that the frontal cortex processes music prior to the auditory‐motor synchronization of music within the motor network. Changes in the pre‐motor cortex appeared last, suggesting that the increase in theta oscillations observed during the intervention was not directly driven by synchronization but was primarily influenced by higher‐order cognitive networks, such as the frontoparietal network. This reveals that the theta‐entrainment process of BWM to the premotor cortex is influenced by top‐down, active perceptual processes in the frontal cortex. Our results confirm that auditory entrainment processes involve the frontal cortex. In this regard, the personalized group significantly outperformed the other three groups. During the BWM intervention, highly synchronized theta oscillatory activity was maintained between the motor cortex and the frontal, parietal, and auditory cortices. This allowed participants in the personalized group to modulate inhibitory control through connections between the premotor cortex and the auditory pathways. Functional magnetic resonance imaging (fMRI) studies have found that this connection can be established through music‐influenced emotional circuits [99]. However, it has also been suggested that this pathway is established through motor circuits influenced by the musical rhythm [100]. In either case, both views emphasize the importance of the frontal brain regions’ active involvement in musical entrainment.

## 5. Limitations

Although our results support the effectiveness of personalized BWM interventions, there remains a lack of investigation into the responses of deep brain regions during this process. Therefore, future studies could use neuroimaging methods (such as fMRI) to explore the activity in deep brain regions during music interventions. In addition, current research focuses primarily on the modulatory effects of inhibitory control. Therefore, this study merely conducted subjective interviews with participants regarding their musical experiences and did not administer specific questionnaires. The participants in the current study were primarily young, healthy volunteers. This group will be expanded in the future to study how BWM affects the inhibitory control function in specific work settings. Additionally, future research could include experiments with participants who have different disorders (e.g., ADHD or Alzheimer’s disease) to provide more references for treating psychiatric disorders with BWM.

## 6. Conclusion

This research demonstrates that personalized BWM effectively enhances inhibitory control through neural entrainment mechanisms. The 10‐min intervention produced measurable improvements in inhibitory control function, mediated by enhanced FM theta activity and strengthened frontoparietal network connectivity. The positive correlation between neural alignment and behavioral outcomes highlights the necessity of personalized approaches in neuromodulation. These findings provide a scientific foundation for developing individualized cognitive interventions, particularly for addressing inhibitory control dysfunction in clinical populations.

## Author Contributions

**Yan Li**: writing – original draft, conceptualization, data curation, investigation. **Xinjian Su**: writing – original draft, conceptualization, data curation, visualization, investigation. **Shenxin Hu**: visualization, conceptualization. **Liju Wang**: data curation, investigation. **Haoyu Bian**: validation, investigation. **Chenxi Qiu**: methodology. **Haohan Yang**: software. **Hua Yang**: resources. **Dezhong Yao**: supervision, funding acquisition. **Jing Lu**: supervision, funding acquisition.

## Funding

This work was supported by the China Manned Space Medical Experiment Project of CMSP (Grant HYZHXMN01005), the Sichuan Science and Technology Program (Grant 2026YFHZ0002), the Brain Science and Brain‐like Intelligence Technology‐National Science and Technology Major Project (Grant 2022ZD0208500), and the National Natural Science Foundation of China (Grant U24A20274).

## Ethics Statement

The experimental protocol was approved by the Ethics Committee of the University of Electronic Science and Technology of China (No. 106142432828160). Participants provided written informed consent before participation.

## Conflicts of Interest

The authors declare no conflicts of interest.

## Data Availability

The data that support the findings of this study are available upon request from the corresponding author. The data are not publicly available due to privacy or ethical restrictions.
